# Decreasing patient-reported burden of treatment: A systematic review of quantitative interventional studies

**DOI:** 10.1371/journal.pone.0245112

**Published:** 2021-01-12

**Authors:** Aurore Lesage, Brice Leclère, Leïla Moret, Clément Le Glatin

**Affiliations:** 1 Department of Medical Evaluation and Epidemiology, Nantes University Hospital, Nantes, France; 2 MiHAR Lab, University of Nantes, Nantes, France; 3 UMR INSERM 1246—MethodS in Patients-Centred Outcomes and HEalth ResEarch (SPHERE), University of Nantes, Nantes, France; University of South Florida, UNITED STATES

## Abstract

**Objectives:**

To describe and analyse studies aiming at quantitatively assessing the impact of interventions on patient-reported burden of treatment as an outcome (primary or secondary).

**Methods:**

The aim of the search strategy was to identify all publications describing a medical intervention intended to reduce patient-reported burden of treatment in adult patients with long-term conditions, from January 1, 2008 to July 15, 2019. Four databases (Medline, PsycINFO, the “Trials” section of the Cochrane-Library, and OpenGrey) were searched in English, French, Spanish, Italian and Portuguese. Each identified article was reviewed and the risk of bias was assessed using a tool adapted from the Cochrane Collaboration recommendations.

**Results:**

Of 641 articles retrieved, 11 were included in this review. There were nine randomized controlled trials, one non-randomized controlled trial, and one before-and-after study. The sample sizes ranged from 55 to 1,546 patients. Eight out of the eleven studies reported significant positive outcomes of the studied interventions. Reducing dosing frequency, improving background therapy, offering home care or providing easier-to-use medical devices were associated with positive outcomes.

**Conclusions:**

Only a few studies have specifically focused on decreasing the subjective burden of treatment. Small trials conducted in patients with a single specific disorder have reported positive outcomes. However, a large, high-quality study assessing the impact of a change in care process in patients with multiple morbidities did not show such results. Further studies are needed to implement this aspect of patient-centred care.

## Introduction

Patient-centred medicine has been advocated for decades by both clinicians and public health specialists [1–3]. In contrast with the biomedical model, patient-centred care requires to “holistically take into account what is known about the patient and understand the patient as a unique human being” [4], which is supposed to improve the quality of interactions between patients and healthcare providers. In this emerging field of research, the burden of treatment is one of the concepts created to describe patients’ perspectives and to be integrated in health policies [5–7].

### Burden of treatment

Initially, the burden of treatment has mainly been evaluated using qualitative methods [8–11], allowing detailed descriptions of the impact of the workload of healthcare on patients’ well-being. The impact has been shown to be much higher and varied than expected. Beside the physical burden (mainly related to treatment side effects) and financial burden (including health expenditures, lost opportunities and the impact on the way of life), patients have also reported a significant impact of time burden (due to numerous medical appointments, daily care, schedule uncertainty), cognitive burden (including all the thinking, learning and planning related to health management) and psychosocial burden (changes in their relationship with relatives and in self-image usually associated with long-term care) [12]. The burden of treatment is also a dynamic concept. Its level may vary over time in a given individual due to the evolution of their psychosocial resources, or as a result of changes in prescribed treatments, regardless of the course of the disease itself [13, 14]. For the same workload of healthcare, the burden of treatment may vary greatly from one patient to another depending on their ability to manage it [15].

### Patient-reported burden of treatment

Given this complex definition, both objective (e.g., number of pills taken per day or healthcare costs in dollars per month) and subjective (e.g., feeling uncomfortable because “the need for regular medical healthcare reminds me of my health problems”, as stated in the Treatment Burden Questionnaire (TBQ) [16]) methods based on patient-reported outcome measures (PROM) are needed to assess the burden of treatment. In 2019, 175 PROM tools dedicated to the burden of treatment have been identified on the specialized eProvide website [17] and most of them were focused on a single disorder. These types of questionnaires are useful but cannot be used to compare the burden of treatment between patients with different diseases, or to assess and follow the disease burden of patients with multiple conditions. Since 2012, several scales have been developed to assess the burden of treatment experienced by individuals with multiple morbidities: the TBQ [18], the Multimorbidity Illness Perceptions Scale (MULTIPleS) [19], the Health Care Task Difficulty (HCTD) [20], the Patient Experience with Treatment and Self-Management (PETS) [21], the Multimorbidity Treatment Burden Questionnaire (MTBQ) [22], and the Medication-Related Burden Quality of Life tool (MRB-QoL) [23]. The burden of treatment is only one of the five subscales assessed in the MULTIPleS, and the HCTD has only been validated in elderly patients (over 65 years), but at least four questionnaires (TBQ, PETS, MTBQ, MRB-QoL) allow an overall analysis of the burden of treatment. They have all been developed in English, and some have also been validated in other languages [16, 24, 25]. Therefore, functional and adapted tools are available [26] to assess the burden of treatment in the population who needs it most, and some researchers agree that decreasing this burden would be a relevant objective [27] to improve patients’ well-being and the efficiency of resource allocation.

### Guidelines mentioning the burden of treatment are missing

Patients with multiple morbidities represent a key public health issue and their management is one of the main challenges that policymakers will have to address over the next years [28, 29]. Multiple comorbidities are also a major cause of high burden of treatment [30], and could impact health expenditures, as they are associated with a lower level of compliance and thus with less effective care [31]. Scientific evidence is thus needed to define healthcare and medical strategies for managing the treatment burden.

Unfortunately, there is no consensus on the appropriate way to manage the burden of treatment in official guidelines, and although many qualitative reviews [32, 33] and papers on policy perspectives are available, little is known about how the burden of treatment is evaluated in quantitative studies, and how it is impacted by medical or pharmaceutical interventions.

That is why we conducted a literature review in order to identify and analyze clinical trials that have assessed interventions aimed at reducing the burden of treatment from the patients’ perspective.

## Methods

### Search strategy

The MEDLINE (through PubMed), PsychINFO, Cochrane Trials section and Open-Grey databases were searched for articles published between January 1, 2008 and July 15, 2019. The search queries are detailed in the appendix. Additional references were requested from experts in the field. Data was collected using a standardized electronic spreadsheet, and screened based on the titles and abstracts. Observational and experimental studies were included if they were published in English, French, Spanish, Italian or Portuguese, and if they used the burden of treatment as a primary or secondary endpoint to assess a long-term intervention that changed the care process in adult patients with long-term conditions (Table 1). Since we focused on the use of PROMs, articles in which the intervention did not significantly change patients’ workload were excluded. For example, for patients treated with insulin, a change in the type of insulin without changing the method and frequency of injections could be completely unnoticed. As most cancers and fertility disorders have time-limited care durations compared to other long-term conditions, we chose to exclude them from our literature review. Finally, studies in which the burden of treatment was evaluated by caregivers, or studies that focused on caregiver burden were considered out of the scope of this study, and thus excluded.

**Table 1:** Inclusion and exclusion criteria. These criteria were used during the screening of abstracts and then full texts.

	Inclusion criteria	Exclusion criteria
**Population**	• Adult (≥18 years) patients with at least one long-term condition	• Caregivers or care providers • Patients on infertility therapy or cancer therapy
**Intervention**		• Interventions that did not significantly change patients’ workload • Surgical interventions
**Outcome**	• The burden of treatment was one of the endpoints • Use of a valid treatment burden measurement tool	• Absence of quantitative assessment of the burden of treatment
**Study design**		• Qualitative studies / Validation studies / Protocol publications / Reviews

The review process was divided between the authors as follows: AL, CL and BL co-developed the inclusion and exclusion criteria; AL applied the inclusion/exclusion criteria to all titles and abstracts; AL and CL reviewed the preselected abstracts and assessed their eligibility; AL reviewed the full texts and CL assessed the citations in case of indecision; AL extracted the data from the relevant articles and regularly discussed the findings with CL and BL.

### Assessment of the burden of treatment

When the burden of treatment was assessed using a single overall score, we considered that an “overall burden of treatment result” was available. When it was assessed using a validated burden of treatment questionnaire composed of several questions (the results of each question being available), we considered that an “overall burden of treatment result and detailed results” were available. Finally, when several questions on the burden of treatment were asked, but no overall result was provided, we considered that the study provided “detailed results only”.

### Assessment of the risk of bias

For each paper, the quality of evidence was assessed using the Cochrane Collaboration risk of bias tool [34]. This tool consisted of seven items rated as *high risk*, *low risk* or *unclear*. When the burden of treatment was a secondary endpoint, the risk of bias was rated for this specific endpoint, and not for the primary endpoint. For information, we also reported the funding details (public or industrial) of the included studies.

## Results

### Study characteristics

**Fig 1:**
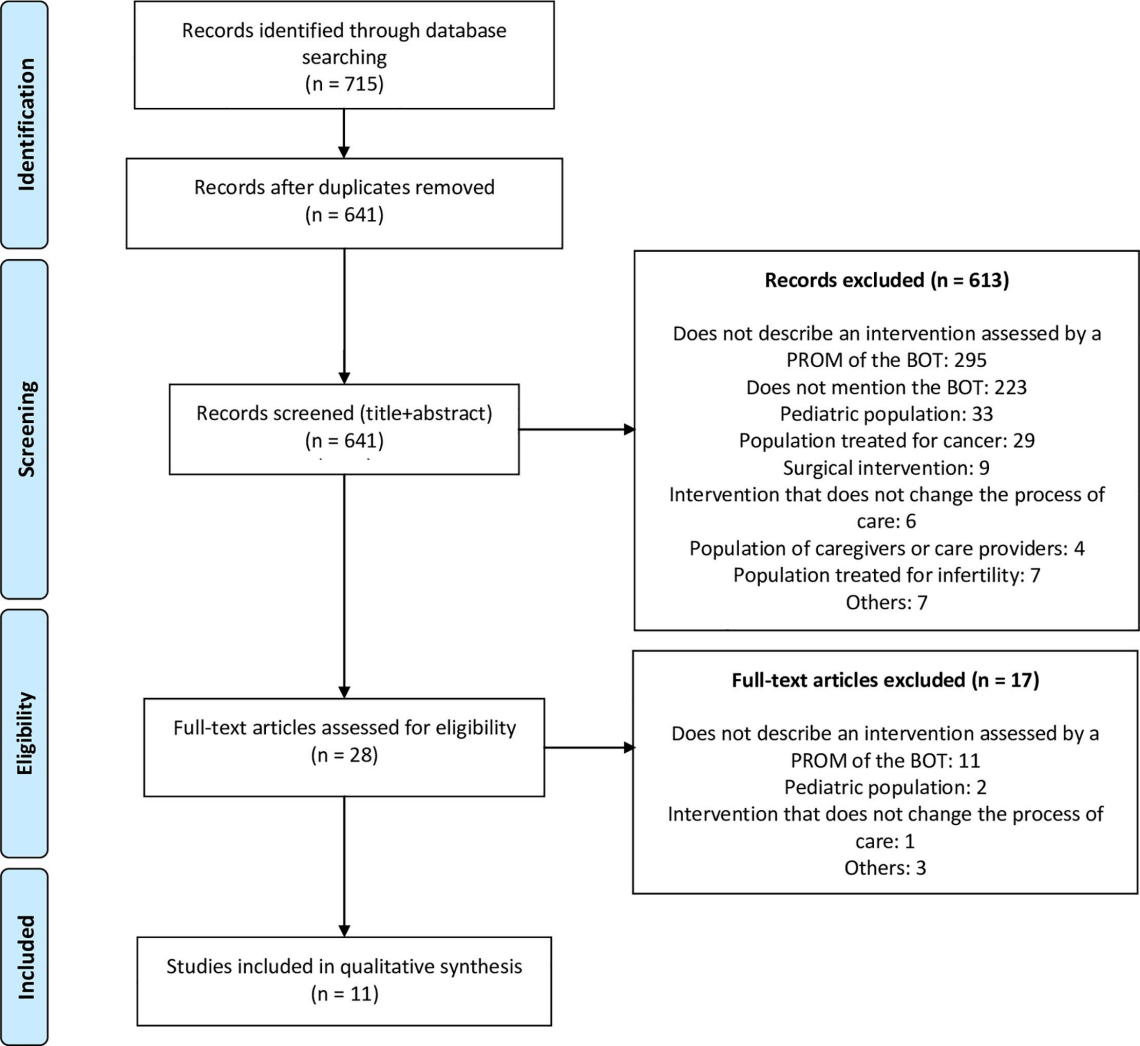
Search flow diagram. BOT = burden of treatment, PROM = patient-reported outcome measure.

The bibliographic search yielded 641 documents (Fig 1). After screening of the titles and abstracts, 28 articles were considered possibly relevant. After screening of the full texts, eleven articles met all the inclusion and exclusion criteria: nine randomized controlled trials, one non-randomized controlled trial, and one uncontrolled before-and-after study (Table 2). Study sample sizes ranged from 55 to 1,546 patients.

**Table 2:** Characteristics of the studies presented in the articles included.

Source	Type of study	Multi- / Monocentric	Country	Number of patients	Medical characteristics	Intervention	Type of intervention	Follow-up duration	Comparator	Primary endpoint
**Reimer (2008)**	RCT	Mono	Germany	60	Patients with type 2 diabetes without any experience with insulin injection devices	Prefilled insulin pen	MEDICAL DEVICE	<1 day	Cartridge insulin pen	Instruction time
							Ease of use			
**Koek (2009)**	RCT	Multi	Netherlands	200	Patients with mild-to-severe psoriasis	Home ultraviolet B phototherapy	CARE	52 weeks	Outpatient ultraviolet B phototherapy	Clinical
							Process			
**Oude Elberink (2009)**	nRCT	Mono	Netherlands	55	Patients with yellow jacket sting allergy (dermal reactions only)	Immunotherapy (VIT) + Epipen	DRUG	52 weeks	Epipen only	Quality of life (PROM)
							Strategy			
**Ishii (2011)**	UBA	Multi	Japan	346	Insulin-naïve patients with type 2 diabetes initiating treatment with BIAsp	BIAsp treatment	DRUG	26 weeks	-	Treatment satisfaction, **including BoT** (PROM)
							Strategy			
**Martin (2013)**	RCT	Multi	-	586	Patients with type 2 diabetes, poorly controlled with insulin glargine, detemir or NPH	Albiglutide once a week	DRUG	52 weeks	Insulin lispro 3 times/day	Biological
							Dose frequency			
**Bilton (2014)**	RCT	Multi	17 countries (Europe and Canada)	302	Patients with cystic fibrosis and chronic infection due to *P*. *aeruginosa*	Antibiotics (liposomal amikacin): 1 inhalation/day	DRUG	26 weeks	Antibiotics (tobramycin): 2 inhalations/day	Clinical
							Dose frequency			
**Quittner (2015)**	RCT	Multi	USA	152	Patients with cystic fibrosis and the G551D-CFTR mutation	Ivacaftor	DRUG	52 weeks	Placebo	Clinical
							Strategy			
**Garg (2016)**	RCT	Multi	USA	242	Diabetic patients with a previous experience with vial/syringe treatment, and without experience with insulin pen	New prefilled insulin pen	MEDICAL DEVICE	24 weeks	Original prefilled insulin pen	Preference for a device
							Ease of use			
**Kabul (2016)**	RCT	Multi	USA	325	Patients with type 2 diabetes, switching from U-100 insulin to U-500 insulin	Insulin U-500 twice a day	DRUG	24 weeks	Insulin U-500 3 times/day	Health-related Quality of Life, **including BoT** **(PROM)**
							Dose frequency		Insulin U-100 more often	
**Salisbury (2018)**	RCT	Multi	England, Scotland	1,546	Patients with 3 or more long-term conditions, followed by a general practitioner	3-D approach (patient-centred care) implemented by general practitioners, pharmacists, nurses	CARE	65 weeks	Usual care by general practitioners, pharmacists, nurses	Health-related Quality of Life, not including BoT (PROM)
							Process			
**Ishii (2019)**	RCT	Multi	Japan	218	Patients with type 2 diabetes, previously managed with diet and exercise only, requiring a DPP-4 inhibitor	Trelagliptin once a week	DRUG	12 weeks	DDP-4 inhibitor once or twice a day	Diabetes Therapy-Related Quality of Life (DTR QOL), **including BoT (****PROM****)**
							Dose frequency			

RCT = Randomised Controlled Trial, UBA = Uncontrolled Before-and-After study, BoT = Burden of Treatment, NPH = Neutral Protamine Hagedorn, PROM = Patient-Reported Outcome Measures, VIT = Venom-Specific Immunotherapy, BIAsp = Biphasic Insulin Aspart, DPP-4 = Dipeptidyl Peptidase-4.

Several types of interventions were evaluated in these eleven articles (Table 2): change in medication (7 studies), change in medical devices (2 studies), and change in the care process (2 studies). Three articles [35–37] assessed the burden of treatment as a primary endpoint, and eight as a secondary endpoint (Tables 2 and 3). Six out of the eleven studies were focused on diabetes, including the three studies that assessed the burden of treatment as a primary endpoint. Nine studies had a follow-up duration of at least 24 weeks, and five studies even had a one-year or longer follow-up duration, which was consistent with the studied population (subjects with long-term conditions).

**Table 3:** Assessment of the intervention in the included articles.

Source	BoT as the primary endpoint	Burden of treatment measurement tool	Impact on BoT	Results
**Reimer (2008)**	NO	DETAILED RESULT ONLY	Positive	• ease of carrying around daily (p <0.001) • discreteness for public use (p = 0.018) • effort required to perform injections (p <0.001) • overall ease of use (p = 0.012)
		• dedicated questions in the various questionnaires		
**Koek (2009)**	NO	DETAILED RESULT ONLY	Positive	• treatment method (i.e. radiation modalities): 2 [1.5–2.4] vs 3.2 [2.6–3.8] (p <0.001) • time lost: 1.4 [1–1.8] vs 4.4 [3.8–5] (p <0.001) • ultraviolet B phototherapy: 2 [1.5–2.3] vs 3.6 [3.1–4.2] (p <0.001) • entire treatment (including topic treatments): 2.6 [2.1–3.2] vs 4.2 [3.6–4.8] (p <0.001)
		• Ad hoc Questionnaire: 4 items assessed using a Visual Analog Scale from 0 (low BoT) to 10 (high BoT)		
**Oude Elberink (2009)**	NO	OVERALL BoT RESULT	Non-significant	• improvement with VIT (randomised subjects): 93.3% • improvement with EpiPen (randomised subjects): 41.7%
		• 1 item ranging from 1 to 7 (1–3 = positive / 4–7 = negative)		
**Ishii (2011)**	YES	OVERALL BoT RESULT + DETAILED RESULT	Positive	• Overall BoT result: score from 64.54 to 67.49 (p = 0.041) • Detailed results: 2 questions with p <0.005: “How bothered have you been by the need to adjust the dosing of your medication?”/“not at all” answer from 36.6% to 51.2%, and “How satisfied have you been with the ease and convenience of your diabetes medication”/positive answer from 40.7% to 53.8%
		• “treatment burden” domain of the DiabMedSat (0–100 scale)		
**Martin (2013)**	NO	OVERALL BoT RESULT	Positive	• Overall BoT result: difference in the change from baseline between groups (weekly Albiglutide vs. three times daily insulin): 6.8 (p <0.001)
		• "Treatment burden" domain of the DiabMedSat (0–100 scale)		
**Bilton (2014)**	NO	OVERALL BoT RESULT	Positive	• Not described in the article
		• CQF-R with 1 domain on BoT		
**Quittner (2015)**	NO	OVERALL BoT RESULT	Positive	• Improvement with Ivacaftor by 4.3 vs 1.3 with placebo (p = 0.042)
		• CQF-R with 1 domain on BoT		
**Garg (2016)**	NO	OVERALL BoT RESULT + DETAILED RESULT	Positive	• TRIM-D–BoT domain: mean transformed score: 5.3 (FlexTouch pen better than FlexPen pen, p <0.001) • TRIM-D DQ–"Device bother" domain: mean transformed score: 8.4 (FlexTouch pen better than FlexPen pen, p <0.001) • ITSQ–"Inconvenience" domain: “FlexTouch pen score–FlexPen pen score” difference: 4.6, (p <0.001)
		• "Burden of treatment" domain (6–30 scale) and "Device bother" domain of the TRIM-D (3–15 scale), "Inconvenience" domain of the ITSQ (5–35 scale)		
**Kabul (2016)**	YES	OVERALL BoT RESULT	Positive	• TRIM-D–BoT domain: difference in the change from baseline (three times daily insulin–Twice daily insulin) between groups: 5.03 [95%CI: 0.99–9.06]
		• "Burden of treatment" domain of the TRIM-D (5-point Likert scale)		
**Salisbury (2018)**	NO	OVERALL BoT RESULT	Non-significant	• MTBQ: adjusted difference in means (Intervention group–usual care group): -0.46 (p = 0.49)
		• MTBQ (5-point Likert scale)		
**Ishii (2019)**	YES	DETAILED RESULT ONLY	Non-significant	• DTR-QOL–“burden on social activities and daily activities” domain: difference in the change from baseline (Trelagliptin–daily DPP4-inhibitors) between groups: 1.938 (p = 0.4536) • DTR-QOL–“anxiety and dissatisfaction with treatment” domain: difference in the change from baseline (Trelagliptin–daily DPP4-inhibitors) between groups: 4.160 (p = 0.896)
		• “Burden on social activities and daily activities”, “anxiety and dissatisfaction with treatment” domains of the DTR-QOL (7-point Likert scale)		

95% confidence intervals are presented within brackets. BoT = Burden of Treatment, VIT = Venom-Specific Immunotherapy, DiabMedSat = Diabetes Medication Satisfaction questionnaire, CQF-R = Cystic Fibrosis Questionnaire-Revised, TRIM-D = Treatment-Related Impact Measure for Diabetes, ITSQ = Insulin Treatment Satisfaction Questionnaire, MTBQ = Multimorbidity Treatment Burden Questionnaire, DTR-QoL = Diabetes Therapy-Related Quality of Life, DPP-4 = Dipeptidyl Peptidase-4

### Assessment of the burden of treatment

The burden of treatment scales used to evaluate the efficacy of the intervention were a general purpose scale (MTBQ) in one article [38], and disease-specific scales in ten studies: two used the Cystic Fibrosis Questionnaire-Revised (CFQ-R) [39, 40], two used the Treatment-Related Impact Measures for Diabetes (TRIM-D) [36, 41], two used the Diabetes Medication Satisfaction questionnaire (DiabMedSat) [35, 42], one used the Diabetes Therapy-Related Quality of Life (DTR-QoL) questionnaire [37], and two used *ad hoc* questions [43, 44]. For some scales, a higher value corresponded to a higher burden, whereas for others, it was the opposite. The scoring systems varied widely, ranging from 0–100 to 1–4 (Table 3).

### Impact of the intervention

Eight, two and one studies reported respectively a significant positive impact, a non-significant positive effect, and no effect of the intervention on the burden of treatment. Five studies provided detailed results on the changes in the dimensions explored by the PROMs, including 4 out of the 8 studies that reported a positive impact (Table 3).

Among the seven studies that investigated a drug-based intervention, four assessed whether an intervention decreasing dosing frequency could have a lesser impact on patients’ lives [36, 37, 39, 42]. Significant positive results were found in three studies, two of which were conducted in diabetic patients. For example, in the study by Martin et al. [42], the “treatment burden” domain of the DiabMedSat improved from 83.3 to 85.5 in the intervention group (injectable drug: albiglutide given once a week) while it worsened from 84.4 to 79.7 in the control group (insulin lispro given twice daily). Three other studies investigated the impact of adding a new drug to improve control on disease evolution, and significant positive results were found in two of them [35, 40, 44]. For example, the study by Quittner et al. [40] showed that adding a gene-based therapy (Ivacaftor) to the background treatment in patients with cystic fibrosis improved the burden of treatment in 44% of patients versus 22% in the placebo group.

Two of the included articles were focused on the potential impact of using simplified medical devices, as a solution to reduce treatment burden: both trials showed a significant improvement in the burden of treatment in diabetic patients using insulin pens [41, 45]. For example, in the study by Reimer et al. [45], a prefilled insulin pen (FlexPen pen) was preferred to a reusable pen and significantly reduced the burden of treatment, as it was considered easier to carry around daily, more discreet for public use, less burdensome to perform injections and overall easier to use.

In the study by Koek et al. [43], another strategy was assessed to reduce the impairment of patients’ lives: they showed that allowing patients with psoriasis to receive phototherapy at home could significantly reduce the reported burden of treatment (differences in mean scores ranging from 1.23 to 3.01 depending on the domain).

In 2018, Salisbury et al. [38] published the results of a study aiming at decreasing the burden of treatment in multimorbid patients, without focusing on a specific disorder. The practitioners were randomized to either providing usual care or using the 3D approach (Dimensions of health, Depression and Drugs), a patient-centred strategy designed to improve continuity, coordination, and efficacy of primary care by implementing a comprehensive multidisciplinary assessment every six months with patient’s nurse, pharmacist and general practitioner. The 3D approach was perceived positively by the patients: the scores assessing the perception of care more centred on the patient were significantly higher in the intervention group, as well as the number of patients reporting being very satisfied with their care. However, the quality of life, disease burden and burden of treatment scores remained stable with no significant differences between both groups (difference in MTBQ scores: -0.46 [-1.78; 0.86], p = 0.49).

### Assessment of the risk of bias

The risk of bias was assessed for each article (Table 4). As the intervention had to be noticeable by the patient, blinding was often not possible, and nine of the studies were open-label studies. One study [40] used a more complex design to enable the use of a placebo control. Another study [38] was an open-label study but used a blinded analysis to reduce biases. Eight studies were funded by the industry (six showed positive results, two showed non-significant results), two by the public (one showed positive results, one showed negative results) and the funding source was not mentioned for one study (showing positive result).

**Table 4:** Quality and risk of bias assessment.

Source	Randomisation	Control	Blinding	Design of experiments	Cochrane risk of bias assessment tool							Funding
					1	2	3	4	5	6	7	
**Reimer (2008)**	yes	Other treatment	**NO**	Crossover	L	U	H	H	L	L	The efficacy of the injection was not assessed	Industrial
**Koek (2009)**	yes	Other treatment	**NO**	Parallel-group	L	L	H	H	L	L	The severity of psoriasis was not defined as an inclusion criterion	Public
**Oude Elberink (2009)**	partial	Other treatment	**NO**	Parallel-group	H	H	H	H	H	L	Some participants were recruited through advertising campaigns	Industrial
**Ishii (2011)**	**NO**	**NO**	**NO**	**No control**	H	H	H	H	H	L		Industrial
**Martin (2013)**	yes	Other treatment	**NO**	Parallel-group	U	U	H	H	L	U		Industrial
**Bilton (2014)**	yes	Other treatment	**NO**	Parallel-group	U	U	H	H	L	U		Unclear
**Quittner (2015)**	yes	Placebo	participants + care providers	Parallel-group	U	U	L	L	L	L		Industrial
**Garg (2016)**	yes	Other treatment	**NO**	Crossover	L	U	H	H	L	L		Industrial
**Kabul (2016)**	yes	Other treatment	**NO**	Parallel-group	L	L	H	H	L	L		Industrial
**Salisbury (2018)**	yes (clusters)	Other treatment	analyst	Parallel-group	L	L	H	L	H	L	Particular profile of eligible practices: 2+ physicians, 4,500+ registered patients	Public
**Ishii (2019)**	yes	Other treatment	**NO**	Parallel-group	U	U	H	H	L	L		Industrial

1 = Random sequence generation, 2 = Allocation sequence concealment, 3 = Blinding of participants & staff, 4 = Blinding of outcome assessment, 5 = Incomplete outcome data, 6 = Selective reporting, 7 = Other sources of bias. L = low, H = high, U = unclear.

## Discussion

The burden of treatment is a recent concept, and articles suggesting evidence-based options to reduce it are limited. This review provides for the first time an overview of the existing strategies that have been quantitatively assessed. Eight out of the eleven interventions analysed in this review reported a successfully reduced burden by improving the treatment strategy, using simplified medical devices, or offering the possibility of being treated at home. On the other hand, the well-designed study by Salisbury et al. [38], conducted in a multimorbid population at risk of high burden of treatment, failed to show a significant difference after implementing advanced patient-centred care. To better understand how to reduce the burden of treatment, it is essential to understand the reasons for these discrepancies.

### Paradoxical results

Surprisingly, the most comprehensive approach aiming at improving patient overall experience did not effectively reduce patient-reported burden of treatment [38]. Since this is the study with the highest level of evidence among the articles included in our review (Table 4), there are two possible interpretations: the validity of the outcomes of the other studies could be questionable; or the discrepancies could be due to differences in the populations studied or in the outcome measures used.

We should seriously consider the first interpretation, given the high risk of bias that we noted in most studies. Indeed, only one of them was a double-blind study, and the randomisation process was not properly described in seven articles.

However, the positive results reported in these nine studies are consistent with the previous qualitative studies of the burden of treatment: the frequency of medication intake, the ease of use of medical devices, and the workload associated with travels to receive care are reported as factors increasing the burden of treatment [9–11, 32, 33, 46]. Moreover, studies with positive results were conducted in patients with a single disease, while we know that the burden of treatment increases and becomes more complex when patients have to manage several long-term diseases [30, 47]. Also, the disease-specific outcome measures chosen in these studies could have focused participants’ attention on the specific effect of the intervention, rather than on their overall burden of treatment.

### Difficulties in assessing the burden of treatment

Based on these assumptions, the choice of the right tool to be used for assessing the burden of treatment appears particularly important. As these tools were used in different settings in the analysed studies, no direct comparisons were possible.

One of the main questions about this methodological issue is whether to choose a generic or disease-specific tool. A generic tool could be interesting to allow comparisons, but could be less precise and sensitive than a specific tool. More generally, using an overall score gathering different aspects of the burden can mask important differences and limit the interpretation of the results. For instance, the study by Salisbury et al. [38] assessed the burden of treatment using the MTBQ that includes items relating to the number of drugs taken and the number of health professionals involved. The lack of significance of the overall result could be due to these specific items, since the authors reported that participants in the intervention group had more appointments with nurses, and received the same median number of drugs than those in the control group.

However, in the studies showing positive results, no specific pattern was identified with regard to the significantly improved subdomains. Thus, a sub-dimensional difference is an interesting hypothesis to explain some of the differences observed, but this assumption cannot be validated based on our results.

Regarding subdimensions, it should be noted that none of the studies included was specifically focused on the financial dimension. The time burden, on the other hand, was frequently assessed, with interventions aiming at reducing the time required per insulin injection, minimizing dosing frequency in cystic fibrosis treatment, or decreasing the travel time for patients requiring phototherapy sessions. In some cases, the physical burden (side effects) played a lesser role than the time burden. For instance, albiglutide given once a week was better rated in terms of treatment burden than insulin lispro given twice daily, even if more patients experienced vomiting and nausea with albiglutide [42]. The psychosocial burden was also successfully improved with the use of more discreet medical devices [41, 45].

### Limited evidence

The relevance of our results is limited by the small number of articles related to this issue, and by the fact that more than half of them are focused on diabetic patients.

This relatively small number of studies could be explained by the fact that the burden of treatment is still a relatively new concept, introduced around 2012 [47], and that no consensual definition has been proposed. For instance, some researchers have used the term as a way to describe the economic consequences of a treatment on the healthcare system, or the psychological consequences of a treatment for caregivers (and not for patients). Conversely, trials that assessed some aspects of the patient’s burden of treatment (treatment-related anxiety for instance) might not yet directly mention it. The burden of treatment is thus still a new and uncommon endpoint in clinical trials. This could have had an impact on the performance of our search strategy, since, for example, there is no dedicated MeSH (Medical SubHeading) term in PubMed, and defining a search strategy with optimal sensitivity and specificity was quite challenging. To include articles assessing the burden of treatment using the same definition as ours, we chose to limit the search to the last ten years. Although we did our best to limit this selection bias, some relevant articles might not have been included in this review.

To limit the heterogeneity of our literature review, we chose to exclude interventions delivered to patients with cancer and fertility disorders, as well as interventions that did not noticeably change patients’ workload. However, we believe that assessing the burden of treatment would also be relevant in these situations, and we acknowledge that these design choices could have been too restrictive and could have limited the identification of relevant articles.

In addition to these methodological issues, the small number of studies identified could be explained by the fact that the burden of treatment is still an under-recognized concept, barely taken into account in guidelines (although some organizations such as the International Consortium for Health Outcomes Measurement [48], are trying to address it), and, from our point of view, is seldom discussed between patients and physicians. Researchers are thus less likely to use it as a relevant endpoint to assess a therapeutic strategy. Indeed, in our review, only three out of the eleven articles used the burden of treatment as a primary endpoint (Table 2), and the other studies could therefore not have been adequately powered to assess this particular outcome. There are many reasons to develop this field of research, and we hope that the importance of assessing the burden of treatment will gradually become more obvious to the investigators. In fact, this could already be the case in the field of diabetes. Indeed, a noticeable aspect of this review is that five out of the eight studies with positive results were conducted in diabetic patients. This is understandable since diabetes control is a key factor to prevent complications, and a low level of compliance is a major obstacle to achieve it. Many studies have shown that using simplified medical devices helped to improve patient satisfaction, but also biological and clinical outcomes such as the number of hypoglycaemic events or the HbA1c level [49]. This beneficial effect on various levels could explain why we identified a higher number of articles on diabetes.

### Conclusion: Implications for research

This systematic review identified eleven studies assessing the burden of treatment in patients with long-term conditions. Their results are heterogeneous and do not allow drawing formal conclusions. Therefore, we can only stress the fact that more data is needed on how to alleviate treatment burden in patients with long-term conditions. Since the burden of treatment is likely to be higher in these patients and because their number is likely to increase over the next few years, future studies should be focused on patients with multiple comorbidities. Also, studies with longer follow-up durations are needed because in published studies, the follow-up is usually less than one year. Indeed, we need to know if the burden of treatment can be reduced over the long term in this population, and how to achieve it.

Researchers should also investigate new strategies aiming at reducing the burden of treatment. In our review, all the interventions were focused on decreasing patients’ workload. However, improving their ability to manage it could be another solution [13], and it would be relevant to assess its efficacy using quantitative tools.

There are ethical and medical justifications for studying the burden of treatment, but its collective and individual impact on health expenditures [50, 51] could also be a decisive motivation. Wasted resources [52], medical overuse [53], and drug burden due to polypharmacy [54, 55] are all good reasons to attempt to decrease treatment burden, and further studies focusing on the public health consequences of reducing the burden of treatment are also needed. This multidimensional approach, mixing individual and collective perspectives, is necessary to promote the concept of burden of treatment, so that it may be more frequently taken into account by health service managers and planners.

## Supporting information

S1 Fig PRISMA checklist. (DOCX)

S1 Table Search strategy used in July 2019. (DOC)
