# Supplementary material for: Decreasing patient-reported burden of treatment: A systematic review of quantitative interventional studies
Source: PLoS One. 2021 Jan 12;16(1):e0245112. doi: 10.1371/journal.pone.0245112 (PMC7802949; doi:10.1371/journal.pone.0245112)
Supplement: S1 Table — (DOC) [file pone.0245112.s002.doc]

**S1 Table. Search strategy used in July 2019.**

| **Database** | **Search query** | **Limitations** |
| --- | --- | --- |
| **Medline (Pubmed)** | (((treatment burden[Title/Abstract]) OR (medication burden[Title/Abstract]) OR (burden of treatment[Title/Abstract]) OR (burden of medication[Title/Abstract])))  AND  ((diabetes mellitus[MeSH Terms]) OR (heart failure[MeSH Terms]) OR (Pulmonary disease, chronic obstructive[MeSH Terms]) OR (Hepatitis, chronic[MeSH Terms]) OR (HIV[MeSH Terms]) OR (Asthma[MeSH Terms]) OR (chronic disease[MeSH Terms]) OR (comorbidity[MeSH Terms])) | 2008-2019 ; Humans ; English, French, Italian, Multiple languages, Portuguese, Spanish |
| **PsycINFO** | AB ( (burden of treatment) OR (treatment burden) OR (burden of medication) OR (medication burden) OR (burden of therapy) OR (therapy burden) )  AND  ( (DE "Diabetes Mellitus") OR (DE "Chronic Obstructive Pulmonary Disease") OR (DE "Hepatitis") OR (DE "HIV") OR (DE "Asthma") OR (DE "Chronic Illness") OR (DE "Comorbidity") OR (DE "Heart Disorders") ) | 2008-2019 ; all journals ; exact terms |
| **Cochrane Library** | “treatment burden” OR “burden of treatment” [Search All Text] | 2008-2019 ; trials |
| **OpenGrey** | “treatment burden” OR “burden of treatment” OR “pill burden” | (none) |
